# Austrian Women

**Published:** 1891-10

**Authors:** 


### AUSTRIAN WOMEN.

Ladies of high birth are wonderfully capable, owing to their excellent system of education, says a Vienna letter to the *New York Evening Post.* Whatever they may be called upon to do—from cutting a dress to making a salad, they are always ready.

Young ladies with titles and fortunes are sent to famous milliners and dressmakers, where they serve a regular apprenticeship, and remain until perfectly able to cut and make any garment.

An Austrian lady who cannot swim or does not know how to ride well is an exception.

Needle work of every kind, even to the making of lace, is part of every young lady's education. There is no smattering of any thing; whether she learns the piano or to draw, she learns it thoroughly. If she has no talent at all for any art, which is seldom, she lets that art entirely alone. Her pedestrian accomplishments put us quite to shame; her efforts of memory are another source of wonder to us.

The wonderful memory which enables Austrian girls to repeat sometimes the whole of "Paradise Lost," or an entire drama, comes from practice begun in babyhood.

Every day the girl is expected to learn a poem or a page. She often does it while making her toilet : and at last a poem requires but a single reading and it is stowed away in the memory safely. As linguists they are famous. This, too, comes from learning when very young. As the court language is French, learning it is compulsory. Even servants are expected to speak both French and German.

The burgher's daughters will not condescend to the learning of dressmaking and cooking, which the titled lady can do without its reflecting on her social position. And so the young women to whom such knowledge would be of practical benefit are inefficient, while all the ladies at the court have at their fingers' ends the power to do any thing.

The Austrian lady of station is acquainted with every detail of the cuisine. A story is told by Viennese ladies of another, who, having neglected this branch of her education, allowed, at a great dinner party which she gave, two dishes of the same color to be served in succession—a fault for which no excuse could be made.

